# A Personal, Distributed Exposimeter: Procedure for Design, Calibration, Validation, and Application

**DOI:** 10.3390/s16020180

**Published:** 2016-02-01

**Authors:** Arno Thielens, Peter Vanveerdeghem, Patrick Van Torre, Stephanie Gängler, Martin Röösli, Hendrik Rogier, Luc Martens, Wout Joseph

**Affiliations:** 1 Department of Information Technology, Ghent University/iMinds, Gaston Crommenlaan 8, Ghent B-9050, Belgium; peter.vanveerdeghem@ugent.be (P.V.); patrick.vantorre@ugent.be (P.V.T.); Hendrik.Rogier@ugent.be (H.R.); Luc.Martens@intec.UGent.be (L.M.); wout.joseph@intec.ugent.be (W.J.); 2 Department of Epidemiology and Public Health, Swiss Tropical and Public Health Institute, Socinstrasse 57, Basel 4051, Switzerland; stephanie.gaengler@cut.ac.cy (S.G.); Martin.Roosli@unibas.ch (M.R.); 3 University of Basel, Basel 4051, Switzerland

**Keywords:** radio frequency sensor, personal exposure assessment

## Abstract

This paper describes, for the first time, the procedure for the full design, calibration, uncertainty analysis, and practical application of a personal, distributed exposimeter (PDE) for the detection of personal exposure in the Global System for Mobile Communications (GSM) downlink (DL) band around 900 MHz (GSM 900 DL). The PDE is a sensor that consists of several body-worn antennas. The on-body location of these antennas is investigated using numerical simulations and calibration measurements in an anechoic chamber. The calibration measurements and the simulations result in a design (or on-body setup) of the PDE. This is used for validation measurements and indoor radio frequency (RF) exposure measurements in Ghent, Belgium. The main achievements of this paper are: first, the demonstration, using both measurements and simulations, that a PDE consisting of multiple on-body textile antennas will have a lower measurement uncertainty for personal RF exposure than existing on-body sensors; second, a validation of the PDE, which proves that the device correctly estimates the incident power densities; and third, a demonstration of the usability of the PDE for real exposure assessment measurements. To this aim, the validated PDE is used for indoor measurements in a residential building in Ghent, Belgium, which yield an average incident power density of 0.018 mW/m².

## 1. Introduction

Radio Frequency (RF) electromagnetic (EM) radiation is increasingly used in modern day society. The emissions of RF radiation are regulated and limited in order to prevent possible health effects [1]. Besides the previously studied thermal effect of RF radiation [1], epidemiological studies are now being executed to determine possible long-term health effects. Both compliance and epidemiological studies require measurement techniques for assessment of exposure to RF radiation. The devices used to measure exposure to RF radiation are Personal Exposimeters (PEMs) [2,3,4,5,6,7,8,9,10,11]. These are body-worn devices, which are meant to measure the instantaneous incident electric-field strength of RF radiation [3,5]. PEMs have some clear advantages over other EM measurement devices, such as broadband or frequency selective (handheld) EMF probes [12] or a combination of an (isotropic) antenna and a spectrum analyzer [13]. First, they are worn on the body and will thus measure at the same location and time as the subject who is wearing the PEM. Secondly, they can measure simultaneously in different frequency bands. Therefore, PEMs are frequently used in RF exposure measurement campaigns [2,3,4,5,7,8,9,10,11] and a protocol has been developed for the correct use of PEMs for personal exposure assessment [2]. However, these devices are affected by severe measurement uncertainties, in which the uncertainty caused by the presence of the body is an important factor [3,4,5,6,7,8,14,15,16]. This uncertainty is caused by two components: first, the body shields the PEMs from a part of the incident EMFs, which introduces an unknown underestimation of the incident EMFs [3,5,6,14] and an anisotropy in the radiation pattern of the body-worn antennas [3,6,14], second, the *a priori* unknown placement of the PEM on the body of a subject causes an uncertainty as well [17,18].

The uncertainty caused by the positioning of PEMs was investigated for the first time in [17] using both numerical simulations and measurements of the electric fields in a transverse plane of the human body. Variations up to 30 dB (a factor of 10³) in measured power density were found for constant incident field strength. Another numerical study was executed by the same group [18], where these results were confirmed. In [5] numerical simulations were executed using a numerical human body phantom placed in a model for a realistic environment at four different frequencies: 100, 946, 2140 and 2450 MHz. Ten potential locations to wear a PEM on the body were investigated. 50% confidence intervals of 8 dB were observed at 946 MHz. In [19,20] numerical simulations at 900 MHz were executed to estimate the uncertainty caused by placing a PEM on the human body. In both studies a median underestimation of the incident fields was observed [19,20], while 50% confidence intervals of several decibels (for example 13 dB in a stationary urban environment) [19] and 95% confidence intervals of 18.5 dB [20] were reported at 900 MHz. 

In [3], the uncertainty on a PEM’s response caused by the varying incident angle of the EM fields was investigated using measurements in eight different frequency bands, using a PEM worn on a fixed position on a rotating subject’s hip exposed by a constant field strength incident from one direction. All the reported 50% confidence interval values on measurements of the PEM found in [3] are larger than 3 dB and range up to 21 dB. The underestimation of PEMs was also confirmed in [3]. On-body calibration measurements of conventional PEMs in an anechoic chamber for several RF frequency bands were presented in [14]. The results of [14] were in line with [3]. In [3,14] the authors suggested the use of a correction factor to compensate for the underestimation caused by shielding of the body and the use of multiple PEMs on fixed positions of the human body in order to reduce the measurement uncertainty caused by the presence of the human body. In [6,21], this was further investigated in the Global System for Mobile Communications (GSM) downlink (DL) band around 950 MHz (GSM 900 DL) and in [15] in the Wireless Fidelity band around 2450 MHz. In [7] numerical simulations were used to determine the uncertainty of PEM measurements in the 98–2450 MHz range when the devices were placed on different locations on the body. A median underestimation of the incident fields by PEMs and large relative deviations (up to 140%) were reported. The authors of [7] suggested the use of correction factors to compensate for the underestimation, in line with what was earlier proposed in [3,6]. The authors of [8] used both numerical simulations and measurements to determine the statistical distribution of the shadowing effect of the human body on PEM measurements. They concluded that a correction factor alone does not suffice to eliminate all measurement uncertainties caused by the presence of the human body, in line with what was shown earlier in [6,14,15,16,21], and also proposed the use of multiple sensors placed around the body in future studies, such as those employed in [6,14,15,16,21].

A possible approach to reduce the uncertainty caused by the presence of the body is presented in [6,21]. In these studies the concept of a Personal, Distributed Exposimeter (PDE) is proposed. This measurement device consists of different antennas which are distributed on the body and used to detect incident RF radiation. This variation of antenna location on the body allows one to reduce the influence of the body [6]. The PDE is a promising technology to reduce the measurement uncertainty on the incident electric field strength [6,15,16,21], but no systematic (calibration) procedure to design such devices has been developed, up till now. In [6] the design of the PDE is based on numerical simulations without the presence of the receiving antennas on the body, which does not take into account polarization and reflection losses in the antennas and thus deviates from a real setup. Following this work, a PDE on arbitrary locations on the body was constructed, calibrated, and used for measurements of in a real environment in [15,21]. However, in these studies no procedure for the design of an on-body network of antennas was included.

The goal of this study is to, for the first time, present a method for the systematic design, calibration, and validation of an operational PDE for the GSM 900 DL band. This frequency band is chosen because it is one of the frequency bands in which the RF exposure is highest [14]. However, other frequency bands can be considered using the same approach [15]. The PDE will be constructed using textile antennas connected to wearable RF power detection nodes. A novel calibration procedure, which is executed using both numerical simulations and measurements in an anechoic chamber, to determine the on-body positions of the textile antennas and to calibrate the PDE, is presented. Using this procedure the measurement uncertainty due to the presence of the body under realistic exposure can be determined and reduced. For the first time, GSM 900 DL signal measurements using a PDE are executed in a real indoor environment. These measurements are validated using a setup in a real environment.

The methods and materials used for the design of and measurements using the PDE are described in Section 2 of this manuscript. Section 3 presents the results of our study, while these are discussed and compared to the literature in Section 4. Conclusions are presented in Section 5.

## 2. Materials and Methods

The calibration procedure proposed in this study can be executed using either calibration measurements or numerical simulations. Textile antennas designed to receive signals on-body in the GSM 900 DL band are used in both approaches. In the first and second subsections, the properties of the used antennas and receiver electronics are described, respectively. Third, the on-body setup is described. In the fourth subsection, the calibration procedure used for a PDE in a realistic multipath environment is introduced. The numerical simulations used to design the PDE are described in the fifth subsection, while the next subsection provides more information on the calibration measurements executed in this study. The validation measurements and the measurements in Ghent, Belgium, are described in the final two subsections.

### 2.1. Textile Antennas

The GSM 900 DL band (925MHz–960MHz) is covered using an aperture coupled shorted patch antenna made from textile materials [6,14,15,16,21,22]. The antenna is linearly polarized and operates at quarter wavelength length to keep down the overall dimensions, resulting in a size of 11.5 cm × 13.5 cm × 1 cm (width × length × height). The conductive parts of this antenna are fabricated using copper plated nylon fabric (conductivity = 0.18 Ω/sq) and the antenna substrate is a foam material (*ε_r* = 1.16, *tan δ* = 0.01), while the feed substrate is made from aramid fabric (*ε_r* = 1.68, *tan δ* = 0.015). 

**Figure 1:**
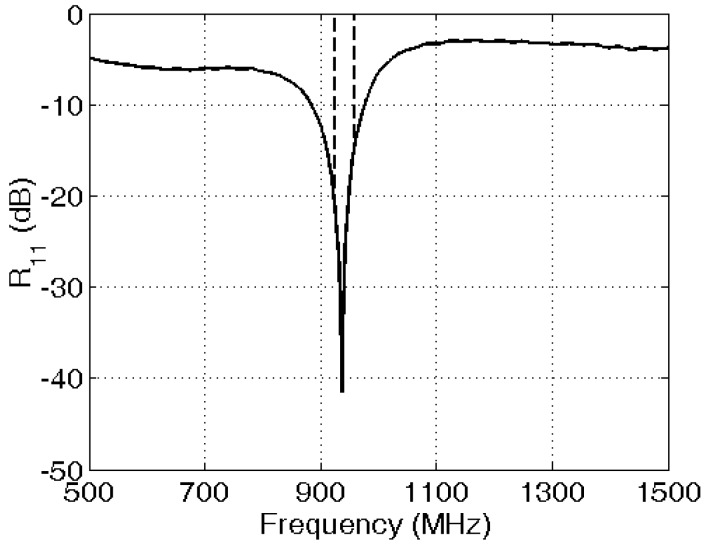
Measured power reflection coefficient (*R_11_*) of the textiles antennas used for the GSM 900 DL band (the band is indicated by dashed lines).

**Figure 2:**
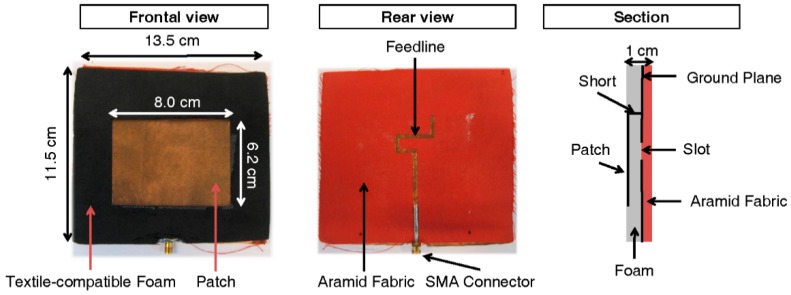
Illustration of the used antennas. More detailed dimensions of the antennas can be found in [22].

An efficiency of 82%, a maximal gain of 3.1 dBi, and a bandwidth of 6.7% ensure good coverage of the GSM 900 DL band. The antenna’s measured power reflection coefficient (lower than −10 dB in the GSM 900 DL band) is shown in Figure 1. An illustration of the antenna is shown in Figure 2, which shows the textile antenna used, its dimensions, and an illustration of a section of the antenna, indicating the different layers. 

The antenna is not designed in this study and was provided by the EM group of Ghent University. Detailed information on the antenna design and performance can be found in [22].

### 2.2. Receiver Nodes for Power Detection

Receiver nodes [21] are developed for the detection of the received powers on the textile antennas. These nodes are connected to the textile antennas using a short (15 cm) SubMiniature version A (SMA) cable (CCSMA-MM-RG316DS-6, Crystek Corporation, Fort Myers, FL, USA). The receiver nodes contain a broadband power detector (1 MHz–4 GHz), placed in series with a band-selective filter tuned to the GSM 900 DL band (part 856528, Triquint Semiconductor, Singapore). Note that the antenna also provides an initial filtering on the incident electric fields, see Figure 1. The different nodes can be powered individually and register received powers on the textile antennas individually, making any interconnection, whether wired or wireless between the different nodes unnecessary. Synchronization of the nodes is ensured by a simultaneous initialization of the nodes at the beginning of the measurements. The receiver nodes sample the received power at an (approximate) sample rate of 1 kHz and calculate statistics of these measurements every second. The following four quantities are calculated by the microcontroller during every second (thus with a sample rate of 1 Hz): the maximal, minimal, geometrically, and arithmetic averaged received powers on the textile antennas. These values are stored logarithmically (in dBm) with an accuracy of 1 dB. The power detection limit of the nodes is determined using an Agilent N5242A PNA-X network analyzer (Agilent, Santa Clara, CA, USA), and is −72 dBm (6.3 × 10^−8^ mW).

### 2.3. On-Body Setup

**Figure 3:**
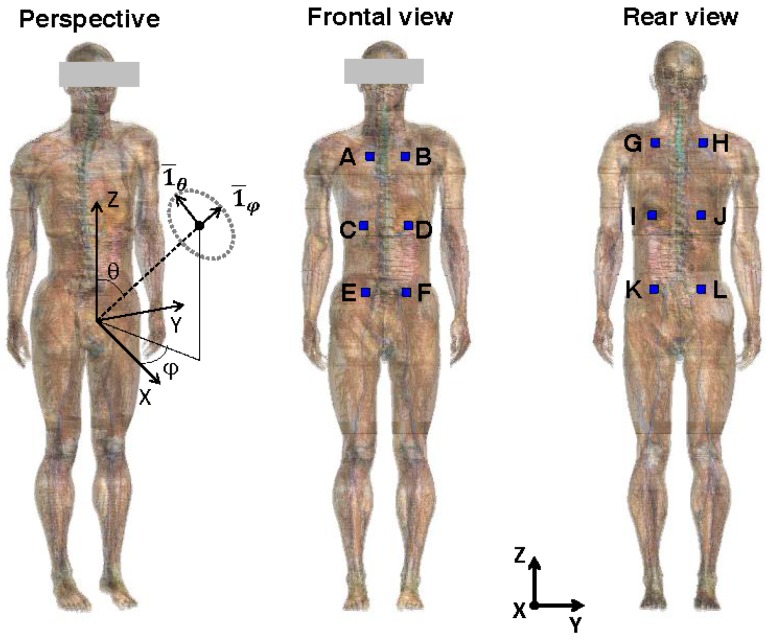
Illustration of the potential locations (A to L) to deploy the antennas on the upper body of the Virtual Family Male.

Twelve possible locations (A to L) to deploy the antennas on the human body are examined, see Figure 3. The limbs, neck, and head were excluded as possible areas for the deployment of antennas, since their movement during measurements would influence the antennas’ performance. Moreover, realistic movements of these body parts cannot be calibrated in the used setup, see Section 2.6. Considering a human in upright anatomical position, only the torso of the human body is considered to position antennas, since the trunk’s orientation remains the same during calibration and measurements. Locations underneath the arms (in anatomical position) are excluded as well. This leaves us with the anterior and posterior sides of the trunk. Both the front and back of the human body are then divided in 6 zones, of which the centers are indicated in Figure 3, where the antennas could be located. These points allow for some separation between the textile antennas (surface = 155 cm²), if they are centered on the indicated locations. In our analysis of combining different antennas (see Equation (11)), combinations where two antennas are placed on the same position are not allowed.

### 2.4. Design Procedure

This subsection describes the procedure used for the design of the PDE. In order to use an antenna as an exposimeter, its on-body aperture (*AA*) has to be determined. In this case the *AA_i_* of the textile antennas on different locations (*i*) on the body, see Figure 2 and Figure 3, have to be determined. This AA_i_ is related to the antennas’ on-body directive gain (*D_i_*) [23]:

(1) $$AA_{i}\left(\theta ,\varphi \right)=\eta_{rad}\left(1-\left|R_{11}\right|\right)D_{i}\left(\theta ,\varphi \right)\frac{\lambda^{2}}{4\pi }$$

with *η_rad_* the radiation efficiency, *R_11_* the antenna’s power reflection coefficient, and *λ* the wavelength.

The *AA_i_(θ,φ)* depends on the elevation and azimuth (*θ* and *φ*), but also depends on the polarization angle (*ψ*) of the incident EMFs. The polarization dependence of *AA_i_(θ,φ,ψ)* can be determined by combining the *AA* for two orthogonal polarizations of the incident electric fields. In this paper, we have chosen to work with the polarizations parallel to the unity vectors ${\bar{1}}_{\theta }$ and ${\bar{1}}_{\varphi }$ in a spherical coordinate system, see Figure 3. The corresponding antenna apertures are denoted *AA_i_(θ,φ,0°)* and *AA_i_(θ,φ,90°)* and can be used to calculate the antenna aperture for any polarization angle (*ψ*):

(2) $$\;AA_{i}\left(\theta ,\varphi ,\psi \right)=AA_{i}\left(\theta ,\varphi ,90{}^{\circ}\right)\cos^{2}\left(\psi \right)+AA_{i}\left(\theta ,\varphi ,0{}^{\circ}\right)\sin^{2}\left(\psi \right)$$

This results in a polarization dependent effective on-body antenna aperture *AA_i_(θ,φ,ψ)* of an antenna placed on position *i* on the body. This *AA_i_(θ,φ,ψ)* provides a relationship between the received power *P_r,i_* on textile antennas *i* and the incident power density (*S_inc_*):

(3) $$P_{r,i}\left(\theta ,\varphi ,\psi \right)=\;AA_{i}\left(\theta ,\varphi ,\psi \right).S_{inc}$$

with *S_inc_* the power density incident from angles *(θ,φ)* with a *ψ* polarization.

Equation (3) can be used to determine the received power from the incident power density (and vice versa) of single incident plane waves. However, in reality multiple plane waves are incident on the antenna. In this case, it does not suffice to simply add the received powers caused by the different incident plane waves, since incident plane waves are able to interfere with each other. The received power should be considered as a function of the incident electric fields:

(4) $$P_{r,i}=\frac{1}{\left|Z_{i}\right|}{\left|\overset{N_{pw}}{\underset{j=1}{\sum }}AF_{i}\left(\theta_{j},\varphi_{j},0{}^{\circ}\right).\left({\bar{E}}_{inc,j}\left(\theta_{j},\varphi_{j},\psi_{j}\right).{\bar{1}}_{\theta }\right).e^{i\alpha_{j}}\right|}^{2}+\frac{1}{\left|Z_{i}\right|}{\left|\overset{N_{pw}}{\underset{j=1}{\sum }}AF_{i}\left(\theta_{j},\varphi_{j},90{}^{\circ}\right).\left({\bar{E}}_{inc,j}\left(\theta_{j},\varphi_{j},\psi_{j}\right).{\bar{1}}_{\varphi }\right).e^{i\alpha_{j}}\right|}^{2}$$

with *Z_i_* the magnitude of the input impedance of antenna *i*, ${\bar{E}}_{inc,j}\left(\theta_{j,},\varphi_{j},\psi_{j}\right)\;$the incident electric field of plane wave *j* with phase *α_j_*, polar angle *θ_j_*, azimuth angle *φ_j_*, polarization *ψ_j_,* and amplitude $\left|{\bar{E}}_{inc,j}\right|$, and *N_pw_* the number of simultaneously incident plane waves. $\;AF_{i}\left(\theta ,\varphi ,\psi \right)\;$is the antennas antenna factor, defined as:

(5) $$\;AF_{i}\left(\theta ,\varphi ,\psi \right)=\sqrt{\;AA_{i}\left(\theta ,\varphi ,\psi \right)\times \frac{\left|Z_{i}\right|}{377}}$$

The usage of the antenna factor in Equation (4) ensures a proper normalization of the incident electric fields according to the directive gain of the antennas.

The number of plane waves and their relative amplitude and phase will constantly change in a real environment. In this paper, the authors will focus on the analysis in three previously studied environments: an “Urban Macro-cell”, an “Indoor Pico-cell”, and an “Outdoor-Indoor” scenario [24,25] in the GSM DL bands. These environments are chosen because they correspond to three investigated scenarios in this paper: outdoor measurements in an urban environment (a garden), indoor validation measurements with a controlled pico-cell, and indoor measurements with an outdoor source. The methods presented in this study could be used for other realistic environments. The scenarios are far-field, dynamic, multipath environments and therefore, the exposure in this environment has to be studied stochastically. To that aim, a large number of exposure samples, consisting of a number of plane waves, are generated according to certain statistics for the properties of the incident plane waves: the number of incident plane waves, and their amplitude, phase, polarization, and polar angle. The distributions of each of these parameters are listed in Table 1.

**Table 1:** Parameters used in the numerical simulations to generate exposure samples in realistic environments (taken from [24]).

	Urban Macro-Cell	Indoor Pico-Cell	Outdoor-Indoor
**Polar Angle** (θ) [26]			
*Asymmetric Double exponential distribution*			
Peak polar angle: $\theta_{0}$(°)	87.8	88.0	90.2
Spread parameter $\theta \;\epsilon \;\left[0,\theta_{0}\right]:\;\sigma^{-}\left({}^{\circ}\right)$	3.9	6.9	5.4
Spread parameter $\theta \;\epsilon \;\left[\theta_{0},\pi \right]:\;\sigma^{+}\left({}^{\circ}\right)$	17.8	9.4	5.5
**Polarization (**ψ)[26]			
*Gaussian distribution*			
Cross Polarization Ratio (dB)	7.3	7.0	10.7
**Nr of Paths (*N_pw_*)** [27]			
*Gao distribution*			
Maximum number of paths: *N_T_*	22	16	21
Distribution parameter: η	2.7	4.7	4.5
**Magnitude E field, Shadowing** [28]			
*Lognormal distribution*			
Standard deviation $\sigma_{E}\left(dB\right)$	6	6	12

The received power on the antennas can be calculated in every exposure sample using Equation (4). Consequently, a distribution of different received powers will be obtained in this environment. These received powers are then normalized to the incident power density, *i.e.*, the sum of the power densities of the different incident plane waves. This results in an effective multipath antenna aperture ($AA_{i}^{mp}$):

(6) $$AA_{i}^{mp}=\frac{P_{r,i}}{S_{inc}}$$

The term $AA_{i}^{mp}\;$can be used to estimate incident power densities from received powers registered on the different antennas i. However, this *AA* is not a constant, but will have a certain distribution, since in a realistic multipath environment the exposure changes constantly and the antenna aperture is different in each exposure situation. From this distribution, the three quartiles: $Q_{i,1}$, $Q_{i,2}$, and $Q_{i,3}\;$are determined by ranking the values of $AA_{i}^{mp}$ and determining the 25% percentile, 50% percentile, and 75% percentile, respectively.

These quartiles can be used to convert measured received powers on textile antennas during measurements in a real environment to incident power densities. The incident power density is estimated as the ratio of the registered received power and the median of the distribution of the antenna aperture:

(7) $$S_{inc,i}^{meas}=\frac{P_{r,i}^{meas}}{Q_{i,2}}$$

where $P_{r,i}^{meas}$ is the power received on a textile antenna placed in zone *i* during a measurement and $S_{inc,i}^{meas}$ is the incident power density estimated using this measurement. The other quartiles of the effective antenna aperture are used to determine the 50% confidence interval (*PI_50_*) on $S_{inc}^{meas}$ [15]:

(8) $$PI_{50}=\frac{Q_{i,3}}{Q_{i,1}}$$

If the *PI_50_* of the distribution of the effective antenna aperture is smaller, then the uncertainty on the measured incident power densities will be smaller as well. In order to reduce this *PI_50_* an averaging over different antennas will be used. The received power on the textile antennas is then averaged over *N* different nodes placed on the body. In this study, we have chosen to work with a geometric average over N nodes:

(9) $$AA_{l}^{geom}=\overset{N}{\underset{k=1}{\prod }}{\left(AA_{k}\right)}^{1/N}$$

with $AA_{l}^{geom}$ the geometric averaged antenna aperture, averaged over the *l*th combination of *N*antennas placed on the body, respectively. This averaged antenna aperture will have a certain distribution, from which quartiles can be obtained: $Q_{l,1}^{geom}$, $Q_{l,2}^{geom}$, and $Q_{l,3}^{geom}$. In this study, the incident power density is estimated using the median value ($Q_{l,2}^{geom}$):

(10) $$S_{inc}^{meas}=\frac{\prod_{k=1}^{N}{\left(P_{r,k}^{meas}\right)}^{1/N}}{Q_{l,2}^{geom}}$$

The *PI_50_* on this incident power density can be calculated with the appropriate quartiles inserted in Equation (8). The goal is to find the combination *l* of *N* antennas which minimizes this uncertainty. To this aim we will use both numerical simulations and measurements in an anechoic chamber.

### 2.5. Design of a PDE Using Numerical Simulations

Finite-Difference Time-Domain (FDTD) simulations are executed at 950 MHz, a frequency in the GSM 900 DL band, using SEMCAD-X (SPEAG, Zürich, Switzerland). First, the textile antenna is modeled in the simulation software. Second, the antennas are placed on a heterogeneous phantom in upright anatomical posture. The heterogeneous phantom used in this study is the Virtual Family Male (VFM, see Figure 3) [29]. This is a three-dimensional human-body model or phantom, based on magnetic resonance images (MRI) of a healthy volunteer. This adult model has a mass of 72.2 kg, a height of 1.80 m and consists of 81 different tissues. The dielectric parameters found in the Gabriel database are assigned [30] to the phantoms tissues, which are discretized in space with a grid step of 1.5 mm in each direction. This phantom is chosen because it morphologically corresponds best, out of the set of available phantoms in the Virtual Family [29], to the subject used in the measurements, see Section 2.6. The antennas are placed centered on 12 points (A to L) located at 1 cm from the front and back of the upper torso, shown in Figure 3. 

The 12 points on the torso are chosen so that they are distributed equidistantly over the full height of the VFM’s torso (from 0.9 m to 1.6 m) and are located at −150° and 150° at the back and, −35°, and 35° (positions C and D) or −45° and 45° (positions A, B, E, and F) at the front of the torso, using the spherical coordinates shown in Figure 3. The textile antennas are placed on the body so that their rear plane does not intersect the phantom (this requires small shifts ≤ 2 cm in the +X or −X directions) and are oriented along the grid, so that their rear plane is parallel to the Z-axis shown in Figure 3.

The textile antennas are placed in two different orientations: vertical (V), with their linear polarization parallel to the Z-axis, or horizontal (H), with their feed line orthogonal to the Z-axis and their rear plane parallel to the Z-axis. The antennas are fed by a voltage source with an impedance of 50 Ohms and radiate during 20 periods, which is long enough to reach a steady-state when the antennas are located in the body.

The on-body directivity of the antennas is then extracted. This directivity can be used to calculate$\;AA_{i}\left(\theta ,\varphi ,\psi \right)$, using Equations (1) and (2). Since the directivity is determined using a numerical simulation, which is exited with one source, all the values of the antenna aperture are in phase. Therefore, they can be used to determine the antenna factors, using Equation (5), that are used in Equation (4). The calibration procedure described above is then followed in order to determine the measurement uncertainty for every combination *l* of *N = 1…12* antennas chosen from 2 × 12 antennas. To this aim, 1000 multipath exposure samples, consisting of N_pw_ simultaneously incident plane waves with phase *α_j_*, polar angle *θ_j_*, azimuth angle *φ_j_,* polarization *ψ_j_*, and amplitude $\left|{\bar{E}}_{inc,j}\right|$, see Equation (4), are generated in order to determine the distribution of $AA_{i}^{mp}$ using Equation (7). A large number of exposure samples (in this case 1000) is chosen in order to obtain stable results for the studied quantities (in this case the quartiles of the distribution of $AA_{i}^{mp}).\;$The calibration is then continued as outlined above and is repeated a 100 times in order to determine the variation on this approach, *i.e.*, determine whether a sufficiently large set of exposure samples is considered.

### 2.6. Design of the PDE Using Calibration Measurements

A setup is constructed in an anechoic chamber consisting of a transmitting antenna (TX), which is a standard gain horn antenna, (NSIRFSG975, Nearfield Systems inc., Torrance, CA, USA) with a gain of 14 dBi at 942.5 MHz and a reflection coefficient lower than −10 dB over the full GSM 900 DL band, and a rotational platform inside the anechoic chamber in the far-field of the antenna. The TX is powered by a Agilent N5242A PNA-X virtual network analyzer at a harmonic frequency of 942.5 MHz (the center frequency of the GSM 900 DL band (925–960 MHz)) with an input power of 10 mW. The incident power densities of the fields emitted by the TX are measured along the axis of the rotational platform as a function of height, using a NARDA broadband probe (Narda NBM-550, Narda Microwave, Hauppauge, NY, USA).

A 26 year old male subject of body mass 82 kg and a height of 1.91 m is placed on the rotational platform in the far-field of the TX antenna. This subject is equipped with a textile antenna and a receiver node placed on one of the 12 zones indicated in Figure 3. The textile antenna is either vertically (V) or horizontally (H) polarized. The subject is then rotated in the azimuth angle φ over 360° with an angular speed of 2°/s. This is repeated for all 12 considered zones, two polarizations of the textile antennas (H and V), and two polarizations of the TX antenna (V and H). The effective on-body aperture $\;AA_{i}\left(\theta =90{}^{\circ},\varphi ,\psi \right)\;$can be determined using the received power on the different antennas and the measured incident power density, see Equation (3). Note that it is not possible using this setup to measure other incident polar angles. Therefore, the analysis of the measured data is continued for *θ* = 90°.

The measurement equipment and calibration setup used do not allow for a simultaneous registration of the phase and the amplitude of the received power on the antennas. Therefore, it is impossible to obtain the correct antenna factor (*AF_i_*) (see Equation (4)) for a constant phase. This phase information is necessary to correctly estimate the effect of interference during multi-path exposure.

The analysis of the distribution of the antenna aperture shall thus be executed using the $\;AA_{i}\left(\theta ,\varphi ,\psi \right)$ obtained in Equation (2). To this aim, 1000 polarization samples *ψ* are generated in the studied environment for every measured (*θ* = 90°,φ) sample. This leads to a distribution of $\;AA_{i}\left(\theta ,\varphi ,\psi \right)$ from which the respective quartiles $Q_{i,1}$, $Q_{i,2}$, and $Q_{i,3}$ are determined. The analysis shown in Equation (9) to Equation (11) can be executed using these quartiles and the $\;AA_{i}\left(\theta ,\varphi ,\psi \right)$ values. This analysis is then repeated 100 times to determine the variation on the analysis. 

In total 12 (positions on the body: A to L) × 2 (polarizations on the body) antenna apertures and their distributions can be determined using the proposed calibration setup and procedure. These antenna apertures are then averaged geometrically over combinations of N antennas drawn from 12 potential locations ($C_{12}^{N}$). This results in a total number of $\sum_{N=1}^{12}C_{12}^{N}\;\times \;2^{N}$ possible combinations of antennas on the body, since two potential polarizations are allowed. The best combinations are then selected for a given number of selected antennas *N*.

### 2.7. Calibration of the PDE

In the previous analysis, using measurements and numerical simulations, only single antennas were used on the body. In order to actually measure with a PDE, we have produced four textile antennas and receiver nodes. In this second calibration step, these are placed on the body of the same volunteer simultaneously and are again calibrated in the anechoic chamber.

The four textile antennas are placed on the subject’s body on the positions and corresponding polarizations that are determined to lead to the lowest measurement uncertainty using the previous calibration step. In this step, an additional constraint is chosen for the placement of the antennas. The PDE has to consist of two H-polarized and two V-polarized antennas in order to minimize the anisotropy of the device. This step is necessary to calibrate differences in the positioning of the antennas between the first calibration and the setup used for measurements. The subject is again rotated in the azimuth angle φ over 360° with an angular speed of 2°/s. During this rotation the receiver nodes will again record the received powers on the four textile antennas. The same procedure, described above, is followed in order to determine the measurement uncertainty of this configuration. 

### 2.8. Validation Measurements in a Real Environment

**Figure 4:**
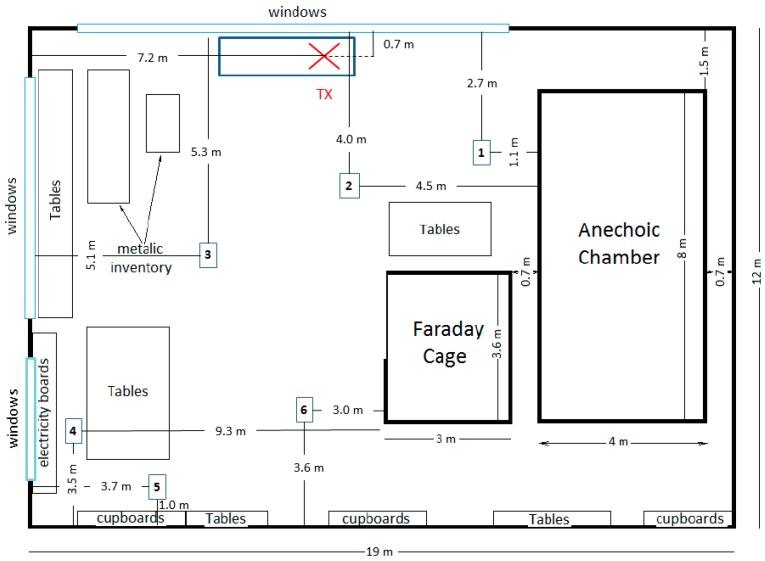
Locations (1 to 6) of the validation measurements and the transmitter (TX) used during the measurements (Courtesy of Marina Marinova).

One can obtain the $AA_{l}^{geom,mp}$ for every combination *l* of a certain number of antennas using calibration measurements or numerical simulations. However, these antenna apertures are based on propagation models, see Table 1, for the environments in which measurements should take place. There are also uncertainties on the assumptions made about the exposure. Therefore, the incident power density that we estimate using the PDE might deviate from the power density measured using an isotropic field probe. In order to determine this deviation, validation measurements are executed on six locations in an indoor environment shown in Figure 4. During these measurements, the “Indoor Pico-cell” and “Outdoor-Indoor” scenarios are validated.

The goal of this validation is to compare the median value measured with a SA (the standard measurement) with the median value measured with the PDE (an alternative measurement device). To this aim, during 60 s, the subject, who is wearing the PDE, walks around in a square of 1.5 × 1.5 m², centered on each of the six measurements points. During these 60 s the room is first excited during 30 s using a pico-cell for use in the GSM 900 DL band (Dual band micro-cell antenna 5027, Jaybeam Wireless/Amphenol antennas, Rockford, IL, USA), which is fed a sinusoidal signal of 19 dBm (79 mW) at 942.5 MHz. This corresponds to the “Indoor Pico-cell” scenario listed in Table 1. During the next 30 s the input power of the pico cell is turned off so all the exposure is caused by outdoor sources. This corresponds to the “Outdoor-Indoor” scenario listed in Table 1. The goal of the movement of the subject during this procedure is to sample the distribution of the effective antenna aperture. A static measurement would not correspond to the used calibration procedure, where a dynamic environment with changing incident fields is assumed. Moreover, during real measurements the orientation of the subject would be unknown. Immediately following these measurements, the incident power densities are measured using a combination of an isotropic, triaxial antenna and a spectrum analyser (R&S FSL, Rhode & Schwartz, Munich, Germany). The spectrum analyzer (SA) measures in the same frequency band as the receiver electronics, using a root-mean-squared (RMS) detector with a resolution bandwidth of 300 kHz. The SA measures in continuous sweep mode and takes 501 points in the studied band, resulting in a sweep time of 0.3 s. The antenna is placed 1.5 m above the ground on each of the six positions and measures the incident power density with and without the pico-cell emitting at 942.5 MHz with the same input power as during the previous measurements. Static measurements with the SA are chosen over dynamic measurements, since they have a lower measurement uncertainty and do not suffer from body shielding of an operator that moves the antenna. The difference between both measurements is then compared in order to determine the deviation between the used models and reality.

### 2.9. Proof-of-Concept: RF Exposure Measurements in a Real Environment

**Figure 5:**
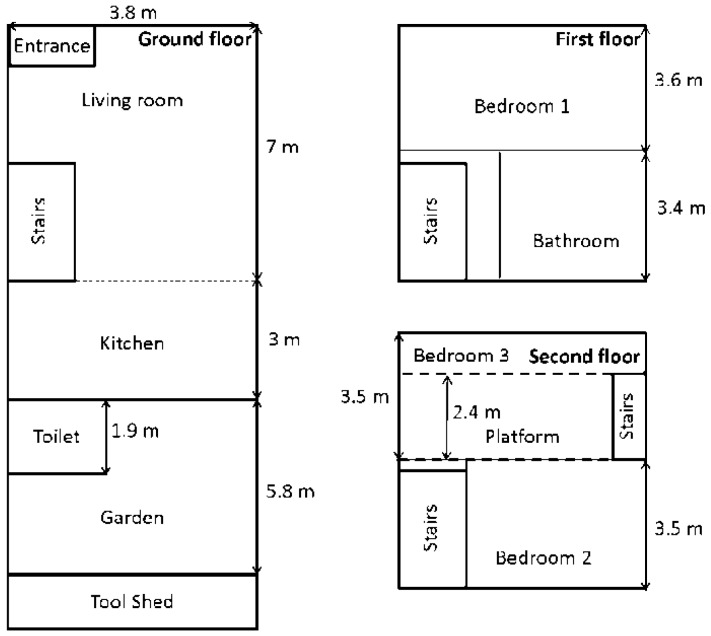
Floor plan of the residential building in Ghent, Belgium, where the RF exposure measurements took place.

Following the calibration, the subject, executes measurements in a residential building in Ghent, Belgium. These measurements correspond to the “Outdoor-Indoor” and “Urban Macro-cell” scenarios. This building has been chosen because it is located on the same route measured in [16], which allows for a comparison. A floor plan of the three story-high building is shown in Figure 5. 

The subject stays 3 min in each room of the house. During these 3 min he walks around (no stationary measurements). Measurements are also performed in the garden of the building. The house consists of a living room, a kitchen, and a toilet on the ground floor, a bedroom and a bathroom on the first floor, and two bedrooms on the second floor. The third bedroom has a platform 2 m above the ground level. The second floor is straight underneath the tiled roof. No GSM emitting devices are present in the house during measurements, so all sources are located outside (base station antennas). The measurements took 1817 s to complete; leading to the same amount of measured average received powers. These powers are converted to S_inc_ values using Equation (11) and the effective antenna apertures determined during the calibration. The indoor measurements are processed using the AA determined in the “Outdoor-Indoor” scenario, while those in the garden are processed using the “Urban-Macrocell” data, see Table 1. Summary statistics are provided for these power densities.Robust Regression Order Statistics (ROS) [2] is not applied to this data, since no censoring occurs due to the low detection limits of the PDE.

## 3. Results

### 3.1. Design Using Numerical Simulations

The $\;AA_{i}\left(\theta ,\varphi ,\psi \right)$ are simulated for every location on body A to L and two polarizations, and are used to calculate the $AA_{i}^{mp}$ for each one of the 24 configurations using 1000 exposure samples in the three scenarios listed in Table 1. This number of samples is associated with a variation of the three quartiles <0.35 dB, determined by repeating the analysis 100 times. The quartiles $Q_{i,1}$, $Q_{i,2}$, and $Q_{i,3}$ are then extracted from these distributions for every configuration *i*. 

**Figure 6:**
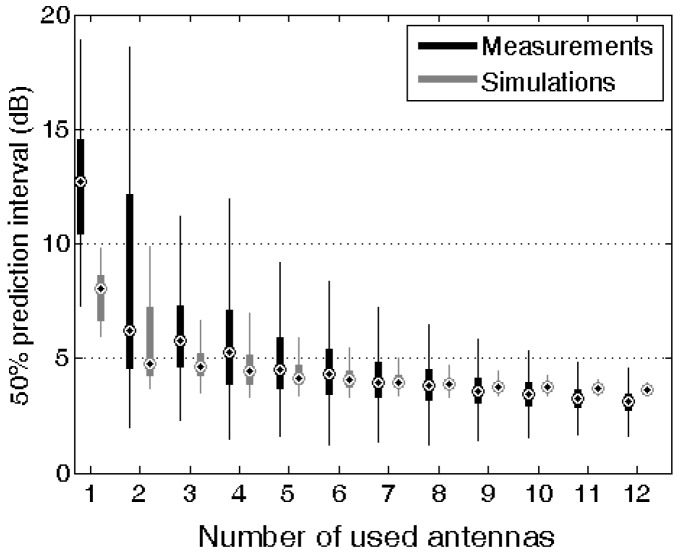
Boxplot of the distribution of the simulated *PI_50_* in the “urban Macro-cell” scenario as a function of the number of antennas. The grey boxes indicate the distribution of the *PI_50_* using numerical simulations, while the black boxes indicate the distribution using calibration measurements. Outliers are suppressed in this figure.

Figure 6 shows (in grey) the distribution of the PI_50_ of the antenna aperture in the “urban Macro-cell” scenario of a combination of N antennas placed either horizontally or vertically polarized on positions A to L, shown in Figure 3. The *PI_50_* decreases with increasing number of antennas. 

The largest reduction in *PI_50_* is found for the situation where a second antenna is added to the first antenna. The single antennas have a median *PI_50_* value of 8.0 dB in the “Urban Macro-cell” scenario. This value is reduced to a median *PI_50_* value of 4.8 dB when averaging geometrically over two antennas, in the “Urban Macro-cell” scenario. Similar values are found in the “Indoor Pico-cell” and “Outdoor-Indoor” scenarios. 

The reduction in *PI_50_* with an increasing number of antennas is explained by two mechanisms: the *1/N²* dependence of the variation on the number of used antennas and a negative covariance between antennas on opposite sides of the body. In terms of variation these dependences can be expressed mathematically as:

(11) $$Var\left(\frac{1}{N}\overset{N}{\underset{i=1}{\sum }}AA_{i}\right)=\frac{1}{N^{2}}\overset{N}{\underset{i=1}{\sum }}Var(AA_{i})+\frac{1}{N^{2}}\overset{N}{\underset{i=1}{\sum }}\overset{N}{\underset{j=1}{\sum }}Cov\left(AA_{i},AA_{j}\right)\left(1-\delta_{ij}\right)$$

with *Var(),* the variance of its input, *Cov()* the covariance of its input, and *δ_ij_*= 1 if *i* = *j* and *δ_ij_*= 0 if *i* ≠ *j*. If the antenna apertures *AA_i_* would be uncorrelated ($Cov\left(AA_{i},AA_{j}\right)$ = 0), then the variation of the average would be the sum of the individual variances divided by the number of uncorrelated responses. This would mean that the variance reduces if the number of used antennas (*N*) increases. In the special case of an average over two uncorrelated sensors that have a response with the same variance, which is not the case here, the variance would decrease with a factor of 2. In case of negatively correlated responses ($Cov\left(AA_{i},AA_{j}\right)$ < 0), then this reduction in variance is even higher, because of the negative term in Equation (11).

**Figure 7:**
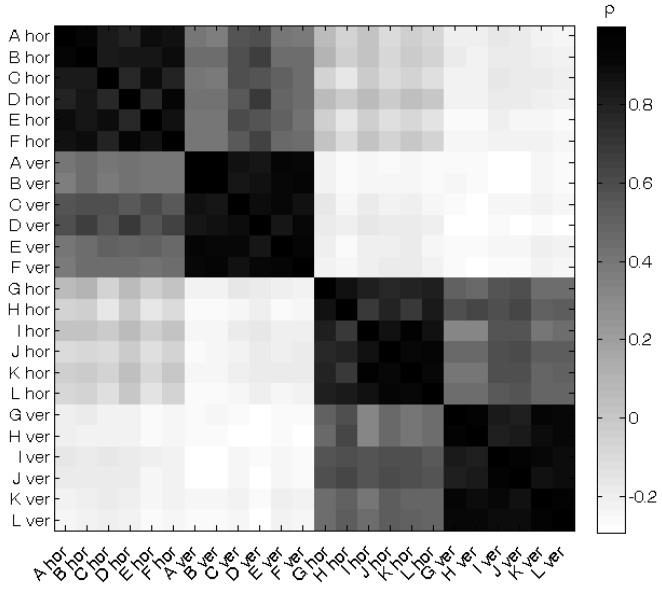
Correlation coefficient of the multipath antenna apertures in the “Urban Macro-cell” scenario for the simulated textile antenna placed horizontally (hor) or vertically (ver) on positions A to L on the VFM.

Figure 7 shows the correlations (ρ) between the antenna apertures for one of the bootstrap sample sets used to determine the distribution of the antenna aperture in the “Urban Macro-cell” scenario. A darker shade of grey indicates a higher correlation coefficient. The main axis of the matrix is black since identical positions and orientations provide perfect correlations (ρ = 1). Further away from the main axis of the matrix, the values are closer to zero (lighter shades) or even negative. The correlations between the antennas on the same side of the body are positive (darker shades of grey), between 0.66 and 0.95 for the co-polarized case and between 0.38 and 0.70 in the cross-polarized case, while the correlation between antennas on opposite sides of the body is predominantly negative (lighter shades of grey), between −0.29 and 0.09 in the co-polarized case and between −0.29 and −0.16 in the cross-polarized case. The largest reduction in variation will thus be obtained by combining antennas on opposite sides of the body with orthogonal orientations. Note that there are positive correlations between horizontally polarized antennas on the front and back of the VFM. For example, the correlation between horizontally polarized textile antennas placed on position D and I is 0.041.

Equation (11) also shows than when two positively correlated responses are combined ($Cov\left(AA_{i},AA_{j}\right)$ > 0), the variance might be reduced, due to the increased number of used antennas, but will be less reduced due to the positive covariance term. This is the case when two antennas on the same side of the body are combined (see Figure 7). Since the studied antenna apertures are not normally distributed, we have chosen to work with rank-based quantities, such as the PI_50_, to quantify the variation on the response. The same principles described in Equation (11) for the variance also apply to the PI_50_, but no closed expressions exist for unknown distributions.

### 3.2. Calibration Measurements of the PDE in an Anechoic Chamber

Figure 6 shows box-plots of the measured *PI_50_* values of all different combinations of *N* antennas on the body in the “Urban Macro-cell” scenario for a geometric averaging over multiple antennas. The measurements confirm that the PI_50_ on the antenna aperture is (on average) reduced when multiple antennas are placed on the body. Note that the distribution of the antenna apertures is different from the ones studied using simulations, since during the measurements in the anechoic room, the EMFs can only be incident with a polar angle of 90°. The largest median reduction in PI_50_ is obtained when a second antenna is added to a single antenna: 6.5 dB for geometric averaging, while adding 10 more antennas “only” reduces the median PI_50_ additionally by 3.1 dB. Considering the combinations with the smallest *PI_50_* values: the best single antenna has a *PI_50_* of 7.3 dB, the best pair antennas has a *PI_50_* of 2 dB, while the best combination of 12 antennas has a *PI_50_* of 1.4 dB. The additional benefits of using a large set of on-body antennas are thus relatively small and a limited number of antennas will thus suffice to achieve an acceptably low PI_50_ value. This is in line with the results obtained using numerical simulations.

**Figure 8:**
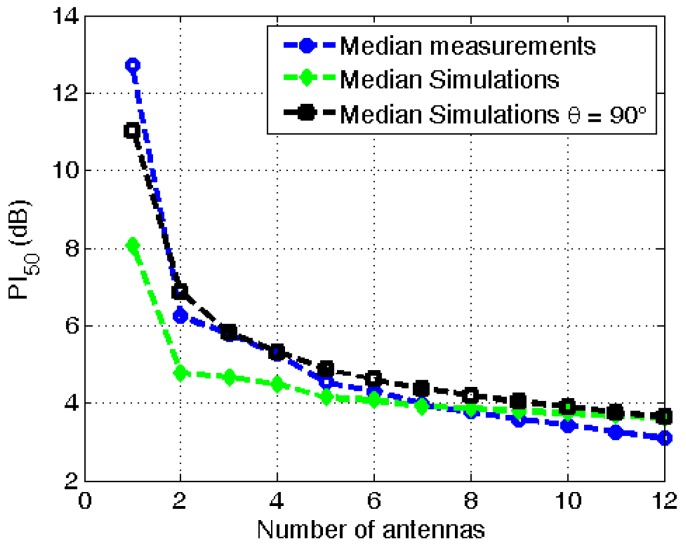
Median PI_50_ on the antenna aperture for a geometric averaging in the “Urban Macro-cell” scenario as a function of the number of antennas using calibration measurements, numerical simulations, restricted to θ = 90°, and the numerical simulations shown in Figure 5.

Figure 8 shows the median *PI_50_* values as a function of the number of antennas using the calibration measurements (blue) and the numerical simulations (black) processed using the same procedure as used for the measurements: restricting the polar angle to 90° and excluding interference of incident fields (no multi-path exposure). Both results are in excellent agreement: for the single antennas the median *PI_50_* differs 1.7 dB, while for higher numbers of antennas the differences are smaller than 0.6 dB.

Figure 8 also shows the median PI_50_ values as a function of the number of antennas in the same environment, for the numerical simulations presented in Figure 6 (green). This is thus obtained using numerical simulations, including interference on the antenna and other polar angles. If combinations of less than six antennas are considered, the numerical simulations including all polar angles predict a lowermedian PI_50_, than the measurements and the simulations that are restricted to *θ* = 90°. This difference decreases for higher a number of antennas; from 4.7 dB for single antennas up to 0.5 dB when 12 antennas are used. The variation caused by interference of incident fields from all polar angles (including other *θ* values than **θ** = 90°), and a varying azimuth angle, cannot be measured using calibration measurements (in the anechoic room). During future calibration measurements, the effect of other polar angles than *θ* = 90°), can be measured by using a transmitting antenna that can be rotated in both azimuth and polar angle around the subject or a spherical array of antennas surrounding the subject. The phase of the received powers (voltages) can be recorded by a vector network analyzer.

The combination of four antennas with the lowest *PI_50_* in the “Urban Macrocell” scenario, under the additional constraint that the set must consist of two H- and V-polarized antennas, is chosen and re-calibrated with all the antennas worn simultaneously on the body. The received powers recorded during the calibration measurements are then processed in order to determine the *AA_geom_* in the three studied environments. The chosen set of four antennas is: two horizontally polarized antennas placed on positions B and G and two V-polarized antennas placed on D and I, see Table 2. The measured mutual coupling (S_21_) between the four simultaneously worn antennas, averaged over the GSM 900 DL band, is smaller than −27 dB.

**Table 2:** Performance characteristics of the selected combination of 4 nodes on the body.

Quantities	Urban Macro-Cell	Indoor Pico-Cell	Outdoor-Indoor
Selected positions^polarizations^	B^H^,D^V^,G^H^,I^V^		
Averaging	geometric		
*Q_i,2_* / $p_{50}(AA_{geom})$ (cm²)	6.06±0.05	5.41±0.06	4.42±0.03
*PI_50_* of *AA_geom_/ S_inc_* (dB)	3.09±0.02	3.71±0.02	3.66±0.01
Detection limit (μW/m²)	0.104±0.001	0.117±0.002	0.143±0.002

Table 2 lists the performance characteristics of the selected combination of antennas in all three studied environments. The median AA_geom_ values for the set of four antennas are between 4.4 and 6.1 cm², given these median antenna apertures and the power detection limit of −72 dBm, a detection limit in terms of the incident power density of about 0.1 μW/m² can be calculated. Commercial PEMs have a detection limit of 0.07 μW/m², which is a factor of 1.5 lower than our detection limit in the “Urban Macro-cell” scenario. However, single PEMs tend to underestimate the incident power density by a factor larger than 1.5 [3,14]. The *PI_50_* values on the median *AA*’s range from 3.1 to 3.7 dB, depending on the environment.

### 3.3. Validation Measurements in a Real Environment

**Figure 9:**
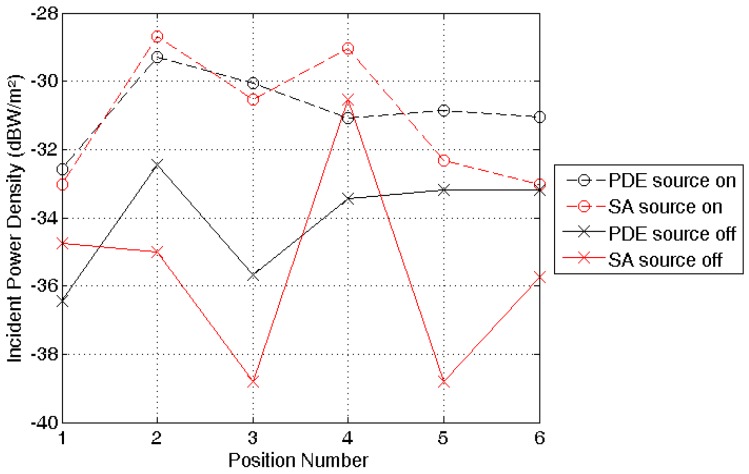
Results of the validation measurements, outlined in Figure 4.

Validation measurements in an indoor environment (see Figure 4) are executed using a PDE consisting of antennas placed on the locations listed in Table 2. Figure 9 shows the median incident power densities measured on the six locations shown in Figure 4 with a spectrum analyzer (SA) and an isotropic antenna (in red) and with a subject equipped with the PDE (in black). 

The values with the pico cell switched on (input power = 79 mW) are connected by dashed lines, while those without a controlled source are indicated by a full line. With the pico-cell emitting at 942.5 MHz, the correspondence between the SA and the PDE is excellent. The average difference between the two measured (logarithmic) power densities is +0.35 dB, which is much smaller than the measurement uncertainties in this scenario. For the measurements without the pico-cell emitting, the correspondence is worse: the average difference is +1.5 dB, with a deviation up to 5.6 dB. 

### 3.4. Proof-of-Concept: RF Exposure Measurements in a Real Environment

**Figure 10:**
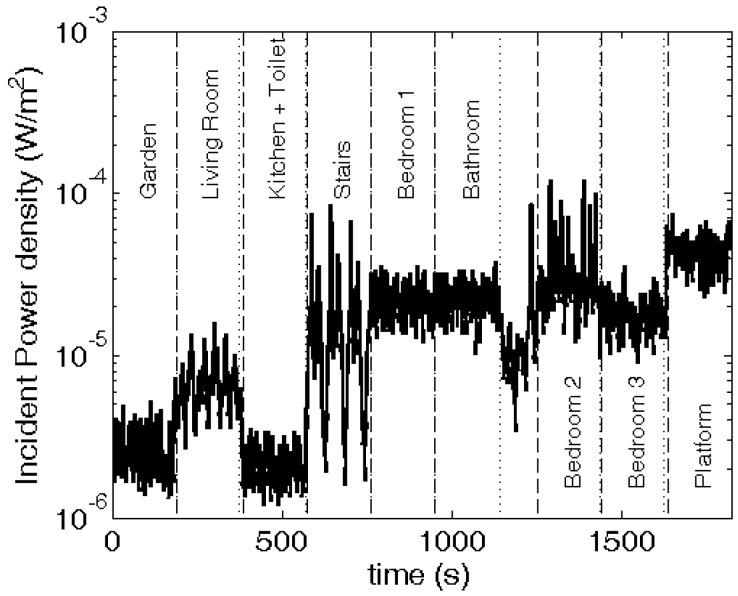
Incident power density measured in the residential building illustrated in Figure 5. The full line indicates measured values with a sample rate of 1 Hz. The vertical lines indicate the beginning and end of measurements in each room.

Figure 10 shows the median measured incident power densities in the residential building shown in Figure 5 and Table 3 lists summary statistics for the different rooms and the garden of the house, which are indicated in Figure 5. The median, measured power density is 18 μW/m² and 95% of the measured samples are lower than 45 μW/m². The highest and lowest measured power densities are 0.12 mW/m² and 1.2 μW/m², respectively. There is a dependency of the measured incident power density on the height in the building. This dependency becomes very apparent when looking at the “stairs” measurements, where the stairs are walked up and down periodically. The highest exposure is measured on the platform in bedroom 2 (right underneath the roof), while the lowest exposure is measured in the kitchen and toilet on the ground floor, inside the house.

**Table 3:** Summary statistics of power densities measured in Ghent in the residential building shown in Figure 5. This table lists the mean incident power density, its standard deviation, and the 25th, 50th, and 75th percentiles of the incident power density in μW/m².

Room	Mean (μW/m²)	Std (μW/m²)	p_25_ (μW/m²)	p_50_ (μW/m²)	p_75_ (μW/m²)
Garden	2.5	0.86	1.9	2.2	2.8
Living Room	6.5	2.5	4.8	6.0	7.6
Kitchen + Toilet	2.1	0.50	1.8	2.0	2.4
Stairs	14	13	4.5	11	17
Bedroom 1	22	5.5	18	20	25
Bathroom	22	5.0	19	23	25
Bedroom 2	30	18	20	24	32
Bedroom 3	18	4.4	15	18	21
Platform	43	9.1	36	43	48
Total	18	15	4.1	17	24

## 4. Discussion

This section discusses the results presented in the previous section: first, the calibration and design procedure of the PDE is discussed, second, a comparison with literature is provided, third, the two different approaches of determining the on-body locations of the PDE are compared, fourth, the validation measurements are discussed, fifth, the measurements in a residential building in Ghent are analyzed, and finally, an evaluation of the properties of an adequate PDE are provided.

### 4.1. Calibration and Design of the PDE

In this study, a PDE for the GSM 900 DL band was designed using numerical simulations and calibration measurements in an anechoic chamber. Using both approaches, the distribution of antenna apertures of different combinations of antennas on the body can be determined. The variation on this distribution is characterized by the *PI_50_*. Both techniques show that these *PI_50_* values decrease as a function of the number of used antennas on the body, see Figure 6 and Figure 8. The calibration measurements correspond very well with the numerical simulations, if both are processed in the same way. If the influence of a polar angle of the incident plane waves and interference of those waves on the antennas is included in the processing of the simulations, then larger differences between simulations and measurements are observed. In this study, we have opted to use a stochastic approach to determine the distributions of the effective on-body antenna apertures. These could also be obtained using the mean effective gain approach [31]. A future study could compare both approaches.

The median reduction in *PI_50_* when adding more antennas to a set of antennas, is larger for the distributions of the AA determined using calibration measurements, than the reductions obtained using numerical simulations. This is explained by the radiation pattern of the used antenna, which has its largest front-to-back ratio in the plane corresponding to *θ* = 90°. This leads to a more negative correlation between the antennas on different sides of the torso, since *θ* = 90° during the calibration measurements.

Following the calibration measurements, the set of four antennas consisting of two H-and two V-polarized antennas with the lowest *PI_50_* value was calibrated again in the anechoic chamber. This set of antennas is used for measurements in a real environment and validation measurements. The *PI_50_* value of this configuration is between 3.1 and 3.7 dB, depending on the environment. This value is a measure for the uncertainty caused by the human body and is low compared to the values found for single antennas. For the single antennas, which are either H- or V-polarized on positions A-L, the measured minimal and median *PI_50_* values are 7.1 and 12 dB, respectively.

A limitation of the calibration measurements is that realistic movement of the limbs is not included, whereas during real measurements the subject’s limbs will move. The calibration and measurements procedure could be improved in a future study, by including several postures during the calibration and using inertial sensors to track and compensate for movement of the limbs [32].

### 4.2. Comparison with Literature

In [14] the *PI_50_* on the on-body response of two conventional PEMs are determined using calibration measurements. The measured *PI_50_* values in the GSM 900 DL band are 8.2 dB, 9.5 dB, and 4.8 dB,for the right hip, left hip, and an arithmetic average over both hips, respectively. In [3] the *PI_50_* of a conventional PEM is determined using calibration measurements. In the GSM 900 DL band, values of 6.5 dB and 15.5 dB were measured for horizontally and vertically polarized incident waves, respectively. In [5], a *PI_50_* on the measured S_inc_ of 8.0 dB was found using numerical simulations in the same frequency bands. For a previous prototype of the PDE using three RF nodes, presented in [6], we obtained a *PI_50_* of 4.5 dB. The listed PI_50_ values in Table 2 (3.1–3.7 dB) are lower than all these values. This indicates that the PDE, presented here, can be used for measurements of the incident power density with less uncertainty than the currently exisiting techniques.

In previous studies, PEMs worn on the body are modeled using points on [5,6,19,20], a surface near [15,17], or a volume near [7] the body of a phantom. These approaches are mainly used because an exact EM modeling of a commercial PEM with an unknown antenna is impossible. In our study we have used numerical models for the used textile antennas. This has the advantage that the actual on-body radiation pattern of the antennas can be considered. The disadvantage is that information about the magnitude of the on-body field strengths is lost. Previous studies were the usage of single on-body antennas, whether they are conventional PEMs [5,7,8,17,18,19,20] or wearable antennas [6,15,16,21], is studied, all conclude that the measurement uncertainty of this on-body configuration is relatively large. In this study, we have used multiple antennas placed on the body, which has the advantage that using this setup, the measurement uncertainty of the PDE can be reduced. The disadvantage is that single band antennas are used, whereas conventional PEMs use a broadband antenna which can measure in multiple frequency bands. A future study should investigate whether multi-band wearable antennas can be employed to design a PDE which measures in multiple RF frequency bands.

### 4.3. Comparison of both Approaches for the Design of the PDE

In this study two different methods are presented to calibrate a PDE on the body of a subject. Both methods have their advantages and disadvantages. The advantages of using numerical simulations compared to using calibration measurements are:

- *Reproducibility*—The placement of the textile antennas on the body is exact and perfectly reproducible. In a calibration in an anechoic chamber, there are inevitable deviations in the on-body placement of the antennas, whereas in numerical simulations there are no deviations between different antennas or receiver electronics. The subject is always in the same pose, whereas a real subject cannot stay perfectly stationary during calibration measurements.
- *Three-Dimensional Gain*—The three-dimensional directive gain of the on-body antennas can be assessed with very small resolution using numerical simulations. The effect of polar angle on the incident plane waves can thus be included. Measurements in the anechoic chamber only provide an antenna aperture in the azimuthal plane and cannot perfectly include the effect of polar angle of the incident plane waves.
- *Phase Information*—The numerical simulations allow one to control all the aspects of the electromagnetic fields and thus also the phase. This allows one to determine the antenna apertures for a constant phase, which is important if the multipath antenna aperture is studies, see Equation (4). In contrast, one is only able to register polarization and amplitude information with the proposed measurement procedure.
- *Adaptability of the Antennas*—The antennas can be adapted and tuned so that they function optimally when used-on body, whereas this approach would be very time and material consuming in reality.

The advantages of using measurements compared to numerical simulations are:

- *Real Setup*—Both the antennas and the subject are the actual subject and antennas used for the measurements, while the phantom and the antennas used in the numerical simulations are mere models for reality. Deviations from the optimal set up such as small displacement or rotations of the antennas are automatically taken into account during the calibration, whereas these can never be modeled exactly using FDTD. Moreover, the measurements are also not faced with uncertainties on the FDTD simulations [33], although there are uncertainties on the calibration as well [34].
- *Adaptability to other Subjects*—Once the antennas are designed and a certain set of antenna positions is chosen on-the body, multiple subjects can be calibrated relatively fast. Whereas using numerical simulations either a phantom of the other subjects should be made using MRI and a labor-intensive process of converting the MRI images to a usable phantom or multiple existing phantoms, for example the virtual family [29], have to be fully simulated with the antennas placed on the body, in order to only approximate the real antenna aperture.

In this paper, we have chosen to work with the results of the calibration measurements mainly because of the first advantage of the measurements listed above.

### 4.4. Validation Measurements

Validation measurements with a SA and an isotropic antenna in an indoor environment, see Figure 4, show a good correspondence with PDE measurements, with an average deviation of only 1.5 dB. This confirms the correct calibration of the measurement device. The correspondence between SA and PDE measurements is better in the “Indoor Pico-cell” scenario than in the “Outdoor Indoor” scenario. A larger deviation was to be expected for the “Outdoor-Indoor” signal, since both the uncertainty due to traffic on the channel and the temporal fading [35] are larger in this case, which increases the measurement uncertainty of both the PDE and the SA. Moreover, we have full control over the dominant exposure in case of the emitting pico cell, which increases reproducability. The best correspondence is obtained in the “Indoor Pico-cell” scenario on positions 1 to 3, which are located closest to the source. This was expected since closer to the source the exposure scenario corresponds best to our calibration measurements (specular multipath), whereas further away from the source diffuse components, which are currently not included in the calibration, migth become more important [36].

### 4.5. Measurements in Ghent

The incident power density is measured using the PDE in different rooms and the garden of a residential building in Ghent, Belgium. The measurements presented in this study are not relevant to determine a typical exposure in Ghent, but can be used to express statistics for the actual exposure of the subject that performed the measurements. In future studies, a more extensive and orchestrated measurement campaign can be executed with the PDE in order to obtain characteristic exposure values in a certain environment, that are not biased by the shielding of the body or an uncertainty on the position of the antennas.

The mean measured power density in the residential building is 18 μW/m², with a standard deviation of 15 μW/m², see Table 3. The relatively large standard deviation is explained by the differences in exposure in the different rooms of the house. First, the outdoor measurements (garden, see Table 3) result in higher *S_inc_* values than those measured in the adjacent indoor room (kitchen + toilet). This is explained by penetration and reflection losses of RF radiation when entering the building. Table 3 also shows that in the living room on the same floor a higher *S_inc_* was measured than in the garden. The dominant RF source at this height in the building could be located on the front side of the building or more shielding by other buildings could occur at the back of the house. However, we do not observe the same effect on the other floors. Second, the height in the building is important for the RF exposure, see Figure 10. The *S_inc_* is higher on the higher floors in the building. This can be explained by the location of the RF sources, which are in this case GSM 900 DL base station antennas. These are typically installed on a tower or on top of a building. In this case the ten nearest antennas that are publically listed in the register of the Belgian telecom regulator are all located higher than 4 m above ground level, with a majority of antennas (all but one) located above 25 m. This dependency is in agreement with [37], where measurements in the same city and frequency band resulted in an increasing power density from 0 to 30 m above ground level.

The measured power densities for GSM 900 DL are comparable to those measured in previous studies in Ghent. In [38], a 95% percentile of 32 μW/m² was measured in indoor residential buildings in Ghent in the GSM DL band. In this study the 95% percentile of the *S_inc_* is 45 μW/m². A higher value was expected since the PDE does not underestimate the *S_inc_,* while the PEM used in [38] does underestimate *S_inc_* [14]. Moreover, the PDE has a higher sampling frequency, which commonly increases the percentiles higher than the median, if a time averaging during the sampling period is used.

In [4], a lower mean value of 6 μW/m² is measured in the GSM 900 DL band inside homes in The Netherlands using an on-body worn conventional PEM. The corresponding mean value during our measurements is 18 μW/m² in the GSM 900 DL band. The measurements in [4] were executed using conventional PEMs, but compensated with a correction factor, determined in [3].

Outdoor measurements of the *S_inc_* in the same frequency band and area in Ghent are presented in [16], using the same on-body setup (*i.e.*, the same antennas and electronics on the same positions of the torso of the same subject) of the PDE. These resulted in a mean *S_inc_* of 23 μW/m², which is higher than the average of 18 μW/m² measured in this study, but expected since the measurements in [16] are executed completely outdoor.

### 4.6. Properties of an Adequate PDE

In order to obtain a representative exposure pattern during a subject’s everyday life, the PDE should not keep them from executing their daily activities. This could be the case if for example the volunteer cannot go shopping or walk without feeling observed by other pedestrians. This could pose limitations and demands on the design, size, weight, or flexibility of the PDE [39]. Moreover, the practicability when taking the device on and off has to be considered. This should be easily manageable for everyone and without help. The current design of the PDE uses lightweight, flexible antennas and electronics that are worn on the body and thus not impede body movement. A subject can wear the PDE and walk around freely. However, the nodes are visible in the current design, which can be solved by wearing them underneath a sweater or integrating them invisibly in clothing. In the current design, the subject was assisted when placing the different nodes on the body. Integrating them in one piece of clothing, which is possible with the current textile antennas and wearable electronics [22], will solve this problem. A Global Positioning System device can be integrated in the clothing as well [40], which will be necessary to track subjects during measurements. 

For the design of the PDE, it has to be taken into account that personal measurements can be conducted in all seasons, temperatures, and weather conditions. In general, the PDE needs to be very robust. The textile antennas are resistant to small shocks, due to their flexibility. Techniques such as covering the textile antennas with a breathable thermoplastic polyurethane coating can protect the antennas and electronics from water absorption and corrosion [41]. The same technique will make the PDE washable, which is necessary for hygienic purposes.

Most epidemiological studies in the field of radio frequency electromagnetic fields, intent to measure for longer periods exceeding 24 h [42,43], to cover as many activities of an individual as possible. At the same time a sufficient sample rate should be provided. The current battery life of the PDE is not sufficient for a 24 h measurement. However, we are aiming to run a future version of the PDE on rechargeable lithium batteries, which are small in dimension (around 4 × 5 × 0.5 cm³) compared to the textile antennas (11.5 × 13.5 × 1 cm³) and can be integrated behind the antenna, without increasing the total dimension of the node.

## 5. Conclusions

A personal, distributed exposimeter (PDE) for measuring incident power densities in the Global system for mobile telecommunications around 900 MHz downlink band (GSM 900 DL) is calibrated using both numerical simulations and measurements on the body of a real subject. This calibration data is used to estimate the effective on-body antenna aperture in different realistic environments. This antenna aperture can be used to estimate the incident power density from received powers on the antennas. From the calibration method one can conclude that a PDE consisting of multiple antennas will have a lower measurement uncertainty than a single antenna worn on the body. The best studied combination of four antennas worn on the body has a 50% prediction interval on single measurements of the incident power density caused by the presence of the human body of 3.1 dB, whereas for the best single antenna on the body this is 7.1 dB. The PDE has been validated in a real indoor pico-cell environment and shows an average relative deviation of 1.5 dB, which is acceptable, compared to the uncertainty on measurements of the incident power density. The same configuration is then used to measure the incident power density in Ghent, Belgium. An average power density of 0.018 mW/m² is measured. 

## Abbreviations

The following abbreviations are used in this manuscript:

PDE: Personal, Distributed Exposimeter
GSM: Global System for Mobile Communication
DL: Downlink
RF: Radio Frequency
EM: Electromagnetic
PEM: Personal Exposimeter
SMA: Sub-Miniature version A
AA: Antenna Aperture
EMF: Electromagnetic Field
FDTD: Finite-Difference Time-Domain
VFM: Virtual Family Male
TX: Transmitting Antenna
